# Mitigating the Burden of Severe Pediatric Respiratory Viruses in the Post-COVID-19 Era: ChatGPT Insights and Recommendations

**DOI:** 10.7759/cureus.36263

**Published:** 2023-03-16

**Authors:** Khalid Alhasan, Jaffar Al-Tawfiq, Fadi Aljamaan, Amr Jamal, Ayman Al-Eyadhy, Mohamad-Hani Temsah

**Affiliations:** 1 Department of Pediatrics, King Saud University, Riyadh, SAU; 2 Department of Kidney and Pancreas Transplant, King Faisal Specialist Hospital and Research Center, Riyadh, SAU; 3 Department of Specialty Internal Medicine and Quality, Johns Hopkins Aramco Healthcare, Dhahran, SAU; 4 Department of Medicine, Indiana University School of Medicine, Indianapolis, USA; 5 Department of Medicine, Johns Hopkins University School of Medicine, Baltimore, USA; 6 Department of Critical Care, King Saud University, Riyadh, SAU; 7 Department of Family and Community Medicine, King Saud University, Riyadh, SAU; 8 Department of Pediatrics, King Saud University Medical City, Riyadh, SAU

**Keywords:** influenza vaccine, human metapneumovirus (hmpv), ai language model, ai chatbot, pediatric intensive care unit(picu), post-covid sequelae, respiratory syncytial virus (rsv), chatgpt

## Abstract

In the current post-pandemic era, the rapid spread of respiratory viruses among children and infants resulted in hospitals and pediatric intensive care units (PICUs) becoming overwhelmed. Healthcare providers around the world faced a significant challenge from the outbreak of respiratory viruses like respiratory syncytial virus (RSV), metapneumovirus, and influenza viruses. The chatbot generative pre-trained transformer, ChatGPT, which was launched by OpenAI in November 2022, had both positive and negative aspects in medical writing. Still, it has the potential to generate mitigation suggestions that could be rapidly implemented. We describe the generated suggestion from ChatGPT on 27 Feb 2023 in response to the question “What's your advice for the pediatric intensivists?” We as human authors and healthcare providers, do agree with and supplement with references these suggestions of ChatGPT. We also advocate that artificial intelligence (AI)-enabled chatbots could be utilized in seeking a vigilant and robust healthcare system to rapidly adapt to changing respiratory viruses circulating around the seasons, but AI-generated suggestions need experts to validate them, and further research is warranted.

## Editorial

During the COVID-19 pandemic, there was a strict application of preventative respiratory measures with the added advantage of decreasing all respiratory infections and hospitalizations, including respiratory syncytial virus (RSV) infections [1,2]. This probably contributed to declining herd immunity [3]. While respiratory viruses with available vaccines could be avoided with increasing vaccination coverage and the use of booster doses before the first winter after the COVID-19 pandemic, other viruses, like the RSV, metapneumovirus, and rhinoviruses, were doomed to flourish and spread in the communities with waning herd immunity and lack of vaccinations [4].

The rapid spread of these respiratory viruses among children and infants resulted in hospitals and pediatric intensive care units (PICUs) becoming overwhelmed with the tsunami of bronchiolitis [5] in both healthy infants as well as high-risk babies (like preemies and immune-deficient children). This 'tripledemic tsunami' was anticipated to push the resilience of healthcare systems 'significantly' at critical points [5]. Due to reaching surge capacity in several PICUs, many hospitals needed to admit children to adult ICUs, which is the opposite of what occurred during the initial COVID-19 pandemic in 2020 [6,7]. Several proposed models about how adult ICUs could support PICUs in public health emergencies, including tele-ICU, were described [7,8].

Protecting infants from severe RSV infections with active immunization is a long-awaited solution. While passive immunization for high-risk infants, such as premature infants and infants with significant congenital heart disease (CHD or chronic lung disease, is available, supply-chain interruptions during the COVID-19 pandemic in 2020-2021 hindered the optimal delivery of these immunoglobulins to the at-risk population during the RSV surge in 2022. Also, the waning levels of RSV antibodies partially explain the recently observed increase in infants’ hospitalizations with moderate to severe RSV infections. Other contributing factors are lifting preventative infection-control measures as the pandemic slowed down [3]. Therefore, protecting the vulnerable population during any viral infections should be tackled with non-pharmacological interventions, such as avoiding social gatherings and universal masking during such times [9].

The chatbot generative pre-trained transformer, ChatGPT, is an artificial intelligence (AI) chatbot that was launched by OpenAI (OpenAI Inc., San Francisco, USA) in November 2022 [10]. While ChatGPT has had both positive and negative aspects in medical writings [11], still, it has been used previously to generate potential mitigation suggestions [12]. In view of the recent surge in respiratory viruses in PICU settings, we asked ChatGPT, on 27 Feb 2023: “Our PICUs are facing increasing numbers of patients with RSV, Metapneumovirus, and other unknown viruses, what's your advice for the pediatric intensivists?”

ChatGPT generated the following quoted suggestions:

“As a language model, I don't have access to real-time information about current events or conditions in specific hospitals or regions. However, in general, here are some recommendations that may be helpful for pediatric intensivists dealing with bronchiolitis cases with RSV, Metapneumovirus and other unknown viruses:

1- Follow the guidelines of your local health authorities and professional societies. These guidelines are based on the latest available evidence and provide a framework for managing infectious diseases. Stay up-to-date on the latest developments in your area and be prepared to adapt your practice as new information becomes available.

2- Implement infection control measures to prevent the spread of the virus. This includes regular hand hygiene, wearing appropriate personal protective equipment (PPE), and using appropriate cleaning and disinfection procedures.

3- Monitor patients closely for signs of respiratory distress, including oxygen saturation, respiratory rate, and work of breathing. In severe cases, respiratory support, such as mechanical ventilation, may be necessary.

4- Treat underlying conditions that may exacerbate the symptoms of bronchiolitis, such as asthma, allergies, and other respiratory infections.

5- Consider the use of medications, such as bronchodilators and corticosteroids, in selected cases. However, the evidence for the effectiveness of these treatments is limited and they should be used judiciously.

6- Provide supportive care to help patients stay hydrated and manage their symptoms, such as fever and cough.

7- Communicate effectively with families, providing education about the virus, its transmission, and the best ways to prevent its spread. Provide emotional support and answer questions as they arise.

8- Finally, remember to take care of yourself and your colleagues, both physically and emotionally. Dealing with infectious diseases can be stressful and exhausting, and it's important to prioritize your own health and well-being in order to provide the best care for your patients.”

We as human authors and healthcare providers, do agree with these suggestions of ChatGPT, seeking to establish a vigilant healthcare system to rapidly adapt to changing respiratory viruses circulating around the seasons, as this would be beneficial for prompt and efficient response. Several healthcare system strategies applied globally during the pandemic were helpful as platforms to advise stakeholders about suitable control measures to be applied in the various phases of respiratory pandemics and the subsequent times [13].

We have demonstrated how ChatGPT could be utilized to generate medical advice and guidelines, with its ability to analyze vast amounts of databases, and how it can assist healthcare providers in generating recommendations and general guidelines for various medical conditions. However, there are some limitations of using ChatGPT for medical advice, including the potential for biased or incomplete information based on the data it was trained on and the risk of providing inaccurate information that may not take into account individual patient factors or medical history. Therefore, it is crucial to use ChatGPT as a tool to augment, not replace, clinical decision-making and to interpret its recommendations in the context of each patient's individual needs and circumstances. Furthermore, future research is warranted on utilizing ChatGPT and other AI-powered chatbots for ultra-rapid suggestions during infectious disease outbreaks or healthcare emergency crises.
